# Robotic-assisted laparoscopic prostatectomy

**DOI:** 10.1038/sj.bjc.6605341

**Published:** 2009-09-29

**Authors:** N L Sharma, N C Shah, D E Neal

**Affiliations:** 1 Department of Urology, Cambridge Research Institute, Addenbrooke's Hospital, Hills Road, Cambridge CB2 0QQ, UK

**Keywords:** robotic, radical, prostatectomy, review

## Abstract

Prostate cancer remains a significant health problem worldwide and is the second highest cause of cancer-related death in men. While there is uncertainty over which men will benefit from radical treatment, considerable efforts are being made to reduce treatment related side-effects and in optimising outcomes. This article reviews the development and introduction of robotic-assisted laparoscopic radical prostatectomy (RALP), the results to date, and the possible future directions of RALP.

Prostate cancer remains a significant health problem worldwide and is the most common cancer affecting men in the United Kingdom, with over 34 000 cases diagnosed in 2005. It is the second leading cause of cancer-related death among men in the UK and the lifetime risk of being diagnosed with prostate cancer is around one in ten. In contrast, in the United States, it is the third commonest cause of cancer-related death among men and the lifetime risk of being diagnosed is one in six, which largely is the consequence of higher rates of PSA testing and screening.

Conventional treatment options include radical prostatectomy (RP), external beam radical radiotherapy, brachytherapy and active monitoring or surveillance with or without regular biopsy. More recently, focal therapies such as cryoablation and high-intensity focused ultrasound have been introduced, but their oncological effectiveness remains uncertain. Radical prostatectomy is an established and accepted treatment for localised and more recently, for locally advanced prostate cancer. Overall 10-year PSA progression rates after RP are around 30%. Recurrence rates are increased in men with a higher pre-operative PSA, Gleason grade, or tumour stage and if there are positive margins in the pathological specimen.

Surgery can be performed by traditional open surgery, or laparoscopically – with or without robotic assistance. This article reviews the development and introduction of robotic-assisted laparoscopic RP (RALP), the results to date, and the possible future directions. In order to concentrate on outcomes when the learning curve has been passed, we have for the purposes of this review included studies where 500 or more cases of RALP have been reported plus other selected references.

## History of radical prostatectomy

The first perineal prostatectomy was performed by Proust in France in 1901, followed by Young in 1905 in the United States of America who performed the surgery on men with prostate cancer. During the early years, there was significant mortality and morbidity, with some surgeons reporting up to 30% mortality rates. Improvements in technique were made, and Millin's retropubic prostatectomy was used for patients with prostate cancer by Memmelaar and others from the late 1940's. It was not until 1983 that the operation was refined by Walsh who also reported the use of a nerve-sparing procedure to improve post-prostatectomy potency rates. The first laparoscopic RP was performed in 1991 by Schuessler and, as with open surgery, advances have been made to improve outcomes, including development of new technologies.

Despite this, there remains significant morbidity associated with RP, most notably urinary incontinence and erectile dysfunction. In general the results from referral centres are better than outcomes derived from national databases, most likely to be related to different outcomes in high- and low-volume centres. The Prostate Cancer Outcomes Study (part of the Surveillance, Epidemiology and End Results (SEER) program), which reports results from a wider variety of centres, many of them low-volume, showed post-RP continence and potency rates of 48 and 20%, respectively, at 2 years (Potosky *et al*, 2000), whereas single-centre series (as shown in Table 1) show continence and potency rates of around 76–97% and 46–87% at a similar length of follow-up.

The difficulties in comparing such outcomes arise from the different methods of recording and reporting complications, the different lengths of follow-up, and varying definitions. When it comes to proxy oncological outcomes there is also the factor of looking at different types of disease because we know that significant stage migration occurs upon community-based screening programmes (Moore *et al*, 2009).

## Development of the da vinci robotic system

A program was established in the late 1980's in the US to develop a system capable of performing remote surgery for use in warzones. Intuitive Surgical realised the commercial potential, and the da Vinci surgical system was launched in 1999. It received FDA approval for general laparoscopic surgery in 2000. The system consists of an operating console from which the surgeon performs the operation and the robot itself with three (or four) operating arms connected to various surgical instruments. The key beneficial features include up to 12 times magnification, three-dimensional vision, and seven degrees freedom of movement with the instruments.

The first RALP was performed in Frankfurt in May 2000 and the first in the UK was performed at St Mary's Hospital, London, in November 2004. Within the UK, there are currently 14 centres using a da Vinci robotic system for urological surgery.

The system itself costs around £1.5 million to install. Even not taking account of capital depreciation and the cost of the robot, each RP carries a real excess cost of around £1300 owing to the use of disposable instruments. The number of hospitals carrying out RALP worldwide is steadily increasing. In the US RALP accounts for 70% of all RP cases. In Europe, this figure is significantly lower, at 14%, but rising.

## Methods of performing robotic rp

The Vattikuti Institute Prostatectomy (VIP) was first described by Menon *et al* (2003) and incorporated principles taken from laparoscopic and open techniques, reflecting the input of European Urologists to its development (Pasticier *et al*, 2001). The main principles of the VIP include development of the extraperitoneal space, lymph node dissection if indicated clinically (i.e. for intermediate- and high-risk cases), incision of the endopelvic fascia, dorsal vein complex control, bladder neck transection, posterior dissection, control of the lateral pedicles, release of the neurovascular bundles, retrograde apical dissection, division of the dorsal venous complex and urethra, vesico-urethral anastomosis, specimen retrieval, and completion.

As with other forms of RP, nerve-sparing procedures should only be performed when the surgeon is confident of achieving good cancer control. Nerve sparing can be either unilateral or bilateral, depending on stage, grade and volume of disease, and pre-operative erectile function. A number of guidelines have been reported to guide the surgeon as to the likely safety of nerve sparing, including the Partin Tables from Johns Hopkins, the Kattan Nomogran from Memorial Sloan Kettering, and the ‘New York University nerve sparing algorithm’, which takes account of the Gleason score, perineural invasion, and tumour volume in the biopsy specimen (Partin *et al*, 1993; Kattan *et al*, 1997; Shah *et al*, 2003).

## Surgical modifications

Since the initial introduction of RALP, various groups have reported modifications to the original VIP technique. The Van Velthoven anastomosis, consisting of a running suture, was reported for both laparoscopic and robotic prostatectomy in 2003 (Van Velthoven *et al*, 2003), and is now a standard technique used in RALP. Kaul *et al* (2005) first reported the preservation of the high lateral prostatic fascia, in addition to the traditional ‘neurovascular bundle preservation’, which led to improved potency rates, probably because it reduces intra-operative tension on the bundles. However, it risks higher positive margin rates (PMRs) and should be reserved for patients with very-low-risk disease. The avoidance of thermal injury of the neurovascular bundle is general good surgical practice and has been highlighted by many authors, including Ahlering *et al* (2006), with short-term results showing a difference in potency rates between cautery and non-cautery techniques.

The Rocco suture was first described in radical retropubic prostatectomy by Rocco in 2007 (Rocco *et al*, 2007) and has been reported by Tewari *et al* (2008) for its use in RALP. This is a posterior reconstruction to support the urethral sphincter, and it has been used in combination with the Pagano suture, which adds further reinforcement to the posterior bladder neck. Tewari *et al* reported an earlier return to total urinary continence using this with 83% of patients being continent at 6 weeks. However, there has been no randomised controlled trial to support the use of either the Rocco or the Pagano suture, and the benefits which have been shown by them could also represent learning-curve effects.

More recently the use of extended pelvic lymph node dissection has been demonstrated in RALP and was shown to be feasible with respect to both surgical technique and number of lymph nodes removed (Feicke *et al*, 2009). The indications and clinical benefit of this in open, laparoscopic, or robotic-assisted approaches remain unclear, although an increased number of positive nodes are found when this is carried out (Dhar *et al*, 2007).

As more surgeons undergo RALP, it is likely that further modifications will be made, aimed at improving functional outcomes. It is important to recognise that oncological outcomes must be perfected and that the primary aim of any radical procedure for prostate cancer is cancer control.

## The learning curve

Learning curves have been reported for open, laparoscopic, and robotic-assisted laparoscopic prostatectomy. A recent review of LRP showed that the benefit of greater surgeon experience in reducing cancer risk continued up to 750 cases, which suggests that the learning curve for LRP is greater than that for open RP (Vickers *et al*, 2009). There are no randomised data, but one real benefit of RALP would be a reduction in the oncological learning curve, compared with LRP. Case report series suggest that this is the case, but there is variation in how learning curves are reported, although most include operating time, blood loss, and rates of positive margins. The learning curve of RALP is associated with the use of innovative technology, loss of tactile (haptic) feedback, and an entirely novel surgical view. It has been suggested that there are two learning curves in RALP – first, to perform the operation safely and with clear margins, and second, to perform nerve-sparing procedures. Menon *et al* (2002), Ahlering *et al* (2003), and Artibani *et al* (2008) reported that the learning curve with RALP for experienced open surgeons is at least 20 cases, simply to perform the operation safely. Ahlering *et al* (2003) and Menon *et al* (2003) also showed that surgeons with previous experience of open prostatectomy have similar complication rates after 20 cases of RALP, compared with a laparoscopic surgeon after 100 cases of laparoscopic RP, suggesting that the learning curve for RALP is shorter than that for laparoscopic RP, at least with regard to complications. There is a suggestion that previous experience of laparoscopy might actually result in a longer learning curve for RALP than for those with open experience (Zorn *et al*, 2007). Other studies have shown much longer learning curves for RALP, with up to 200 cases (Mikhail *et al*, 2006).

While uncertainty surrounds the duration of the learning curves for RALP, it is critical that centres embarking on starting a program do so with supervision and structured support. Close mentoring by an experienced team is beneficial when starting to perform RALP: Kaul *et al* (2006) reported a two-person mentoring team (with experience of over 1000 RALPs) training the console surgeon and the assistant for five cases, after the entire surgical team had undergone 1 week of intensive training. This was followed by a period of mentoring by an experienced laparoscopic surgeon for a further 40 cases. This approach has led to satisfactory outcomes and such approaches should be used by centres implementing RALP. The optimal duration of this mentoring period is not clear; Dr Menon was proctored for the first 100 cases that he performed. A structured approach when starting this technique should include mentoring by experienced RALP surgeons for as long as is necessary to pass through the ‘learning curve’, in order to offer patients the best possible oncological and functional outcomes. However, a standardised program for the implementation of RALP has not yet been developed. There has been concern that initial outcomes have not been as good as they should have been because of inadequate mentoring. Ideally large-volume centres would provide a ‘mentoring team’ for new RALP centres, with an appropriately targeted mentoring period depending on the surgeon's competence.

## Results of different types of rp

There are no randomised controlled trial data on the outcomes of robotic-assisted *vs* laparoscopic *vs* open RP. Until such a study has been performed, the best observations come from reviews, which have compared outcomes from all three surgical options. Table 1 shows outcomes from large series of laparoscopic and robotic-assisted surgery.

In our institute, more than 440 men have now undergone RALP, with a mean age of 62 years and an average operating time of 180 min. We have a stringent system of pathological reporting with step-sectioning of whole mounted specimens. As with other series, the PMR correlated with pathological stage, with 16% in pT2 and 36% in pT3. Two patients had pT4 disease and both had positive margins. While these stage-specific PMRs for T2 disease are somewhat higher than those from contemporary open and laparoscopic series, this is, in part, likely to represent a difference in disease burden rather than a difference solely due to surgical technique. For instance, 42% of our patients had pT3 disease, indicating a population with higher stage disease than in other centres, particularly those with screened patient populations. When these results are compared to open series from the US before PSA screening took place, the PMRs are much more comparable (around 30% overall, with higher rates for pT3 and pT4 disease; Wieder and Soloway, 1998). More recent comparisons of European countries, where PSA testing is common, with the US have shown that the overall pathological stages are similar (Gallina *et al*, 2008), suggesting that the UK results are related to a low baseline level of PSA testing (Moore *et al*, 2009). However, we recognise that our PMR for patients with pT2 disease needs to be improved, and this is likely to be achieved by improving our patient selection for nerve-sparing procedures. With regards to functional outcomes from our institute, 75% of men are sufficiently potent for intercourse at 12 months, with 25% of these men using no adjuvant treatment. For patients in whom the Rocco stitch was used (the last 200 patients), 80% of men are fully continent at just 6 weeks. At 10 months, 94% are completely continent.

In a recent separate analysis of a single surgeon's learning curve from our institute, we noted continued improvement in surgical outcomes for the last 70 cases: the overall PMR for these later cases is 19% and stage-specific rates are 10, 23, and 100% for pT2, pT3, and pT4 respectively. Median operating time is reduced at 2 h 10 min and median blood loss is 150 ml. These data are comparable with evidence from open surgeons that volume and outcome are related, in other words high-volume surgeons have lower positive margin rates (Chun *et al*, 2006; Wilt *et al*, 2008).

With respect to blood loss, it appears that RALP is superior to open and laparoscopic RP (Frota *et al*, 2008). While early results are promising, some studies have reported higher complication rates with RALP compared with open RP: Hu *et al* (2008) showed an anastomotic stricture rate of 15.2% in the RALP group, compared with 12% in the open group. However, only 608 patients had RALP, compared with 2094 patients who had open RP, and this was not a single-surgeon analysis. These rates of anastomotic strictures have not been found in most studies, and have not been found in the authors’ series.

Despite there being many published studies, there are few and limited comparative data available, due to the differences in outcome measures recorded by different groups, as has been recently highlighted by a review of the surgical options for prostate cancer (Ficarra *et al*, 2009). In order to fully counsel a patient about which surgical modality to choose with regards to complications, and oncological and functional outcomes, a large randomised controlled trial comparing open *vs* laparoscopic *vs* robotic-assisted prostatectomy is required. However, in the meantime centres should provide patients with their own local outcome data.

There are common predictors of recurrence after all forms of RP, and these include high pre-operative PSA, high Gleason grade, high pathological stage, and positive surgical margins. All the series shown in Table 1 highlight the low PMR associated with low-stage disease, that is pT2, but higher rates with higher stage disease. We should aim to reduce the PMRs associated with both pT2 and pT3 disease.

The stage and grade of prostate cancer in different countries varies significantly depending on the underlying rate of prostate cancer screening. It is clear that widespread community-based PSA testing results in ‘stage migration’ where most of the cancers are detected at T1 or T2 disease of Gleason grade 6, with low volumes of cancer on biopsy (Postma *et al*, 2006; Collin *et al*, 2008; Moore *et al*, 2009). Such men will have low rates of positive margins. In the UK, the underlying rate of PSA testing is low, and many more of the cancers detected will be of higher volume and grade, and even in the D’Amico low risk category will tend to have higher volumes of cancer – this needs to be borne in mind when comparing UK figures with those from the US and Europe where screening is more common.

Variations in pathology reporting can also affect the PMR due to differences in specimen sampling. Desai *et al* (2002) reported higher rates of detection of extra-prostatic extension in specimens, which underwent complete embedding and close step-sectioning, compared with partial sampling. Clearly this has implications for higher detection rates of positive margins, and the method used by each centre should be considered when interpreting PMRs.

Rigorous and standardised follow-up data collection is crucial to allow adequate comparison between reported series and assess individual performance. While oncological and functional outcomes are routinely evaluated, quality of life (QoL) is often overlooked. Namiki *et al* (2009) have studied the effect of open RP on QoL using the Short Form 36-Item Health Survey (SF-36) and other prostate-specific questionnaires, and showed that by 5 years most men had returned to baseline levels of QoL. White *et al* (2008) compared QoL outcomes for patients from the CapSURE database with locally advanced prostate cancer undergoing different treatments, and showed that all treatments were associated with reductions in QoL scores, although they were unable to determine the ‘best’ treatment with regards to QoL.

The results of the ProtecT Study will address some of these QoL issues relating to different treatment options (Donovan *et al*, 2002; Wilt, 2008), but larger studies of RALP are required to show whether there is any benefit on QoL as compared with other treatments. If functional outcomes improve earlier after RALP, it may be that patients would return to a baseline QoL sooner following RALP as compared with other treatments.

A recent study by Schroeck *et al* (2008) showed that patients who had RALP were 3–4 times more likely to be dissatisfied and regretful than patients who had undergone open RP. The authors proposed that this was likely due to the higher level of expectations by the RALP patients, which highlights the importance of providing patients with accurate, ‘neutral’, and realistic information regarding post-operative recovery and function. Indeed, this approach has resulted in 91% of RALP patients expressing no dissatisfaction or regrets in the series from the Vattikuti Institute (Menon and Bhandari, 2008). We believe that careful presentation of outcome information and data, coupled with a more neutral approach with respect to discussion of radical *vs* conservative approaches, will result in lower rates of dissatisfaction. This is of particular relevance when introducing RALP, and when a single centre has performed far fewer than 500 cases. In order for informed consent to be obtained, a frank and thorough discussion must take place, highlighting the likely outcomes for the patient based on the surgeon's own data, rather than the frequently quoted large studies in the literature with favourable outcomes.

## Future directions

As improvements in operative techniques continue to improve rates of continence and potency, the robotic system is likely to gain in popularity. In addition, the system itself continues to be updated, with the latest ‘Si system’, including high-definition screens. The loss of tactile feedback remains an issue and novel engineering approaches are being tested to try to resolve this issue. Also there will be a need for continued modifications of the instruments to try to increase their delicacy, such as the need to decrease the size of the bipolar instrument. Due to the significant costs, however, it is likely to remain a system used only by larger centres, and it should be mandatory that continuous review of the outcomes is undertaken by each department. This should be standardised in order to allow comparisons between centres and included in this must be detailed assessment of continence and potency rates (pre- and post-operatively), and complications. These standards should be used by all surgeons performing RP and this will enable a large, prospective, multicentre randomised trial to be performed comparing robotic-assisted, open, and laparoscopic RP. Surgeons will be required to show the benefits of RALP for their own centre, and each surgeon's outcomes must be assessed. It is not appropriate for centres to quote rates of other institutions, which may be far more favourable than their own.

The robotic system has recently been used in conjunction with laparo-endoscopic single-site surgery to perform RP in several institutions (Barret *et al*, 2009; Kaouk *et al*, 2009). This was performed via a single umbilical incision, with all instruments used through the single site. This illustrates the shift in surgery over recent years to provide a procedure with optimal oncological and functional outcomes, with a cosmetic result comparable to that of minor surgery.

It is clear that surgery must be applied to those patients most likely to benefit. Some men with low-risk prostate cancer will not benefit from radical treatment of any sort, but identification of them remains problematic. The approach of active surveillance or active monitoring, where men are followed up carefully with regular PSA testing, is one approach, but not as yet proven to safely identify men at risk of future prostate cancer morbidity (D’Amico *et al*, 2005, 2006). It is likely that a combination of personal genetic information (Eeles *et al*, 2008) coupled with measurement of several biomarkers in serum, urine, and most importantly in prostate biopsy tissue, will be able to identify men at high or low risk of progression, which will focus targeting radical treatment to the right group.

Traditionally surgery has not been widely used for intermediate- and high-risk prostate cancer, but results have shown reasonable long-term cancer control rates (Ward *et al*, 2005; Hsu *et al*, 2007). Indeed there is a growing realisation that surgery should be targeted at these men, although higher rates of positive margins requiring post-operative radiotherapy will be found. Should we be aiming RALP at those requiring management of locally advanced disease where oncological outcomes remain poor but could be significantly improved? Lessons from the management of breast cancer can be learnt, with programmes of multi-modal treatment offered to high-risk cancers. Currently, RALP is used most for patients with low-risk localised disease. It is already widely published that PMRs increase with tumour stage and that positive margins are an independent risk factor for recurrence. If PMR can be reduced by an enhanced surgical technique, then progression-free rates might be improved for a subset of patients with higher risk localised disease. Currently, progression-free rates at 10 years are 37% for patients with pT3b disease, 76% for pT3a disease, and 93% for pT1/T2 disease (Han *et al*, 2001). Given the benefits of robotic surgery, it seems logical to propose that the operation could be optimised for patients with locally advanced disease, and give outcomes which are more favourable than those currently achieved.

## Conclusion

The lack of randomised trials in this new technology makes assessment difficult. However, there is clear evidence from retrospective studies that robotic-assisted laparoscopic prostatectomy can achieve excellent oncological and functional outcomes. Centres which are embarking on this procedure must be aware of its associated learning curve and should adopt a carefully structured mentoring approach to minimise this. Follow-up data should be comprehensive and standardised, and include QoL scores, which will enable results of future randomised controlled trials to be useful in clinical practice. The future of robotic surgery for prostate cancer is exciting and there are likely to be many advances made, including improved outcomes for patients with locally advanced disease. With the increasing use of RALP, it is important for surgeons to counsel patients and provide realistic expectations of the outcomes, including the likelihood of multi-modal treatment, when locally advanced disease is present. Caution must be exerted when ‘developing’ technologies, and it must always be remembered that the primary aim of radical therapy is cancer control.

## Figures and Tables

**Table 1:** Comparison of outcomes of different types of radical prostatectomy

	**Patient**	**Overall**	**PMR by pathological stage (%)**				**EBL**	**Mean operative**	**Follow-up**	**Continency**	**Potency**	**Biochemical**
**Series**	**number**	**PMR (%)**	**T2**	**T3a**	**T3b**	**T4**	**(ml)**	**time (min)**	**(months)**	**%**	**%**	**recurrence^a^**
*Laparoscopic:*												
Lein *et al* (2006)	1000	26.5	15	53	60	100	200	266	29	76	—	9.5
Touijer *et al* (2009)	1564	13.0	—	—	—	—	—	—	96	—	—	29
Eden *et al* (2009)	1000	13.3	—	—	—	—	200	177	28	95	66	3.9
												
*RALP*												
Badani *et al* (2007)	2766	—	13	35^b^	35^b^	—	142	154	22	93	79	7.3
Patel *et al* (2008)	1500	9.3	4	34^b^	34^b^	40	111	105	—	—	—	—
Tewari *et al* (2008)	700	—	—	—	—	—	—	—	12	97	87	—
												
Authors’ institute^c^	440	23	16	30	51	100	200	180	12	94	75	—

Abbreviations: EBL=Estimated Blood Loss; PMR=positive margin rate; RALP=robotic-assisted laparoscopic radical prostatectomy.

a Biochemical relapse rate (usually at PSA >0.2 ng/ml) is reported at various times after surgery making direct comparisons difficult.

b PMR for all pT3 specimens.

c See separate note on recent data from one surgeon.
